# Bone Marrow Abnormalities in HIV Disease

**DOI:** 10.4084/MJHID.2013.033

**Published:** 2013-06-03

**Authors:** Sharad A. Dhurve, Alka S. Dhurve

**Affiliations:** 1 Assistant Professor, Department of Medicine, NKPSIMSR, Nagpur, Maharashtra; 2 Assistant Professor, Department of Anatomy, Indira Gandhi Government Medical College, Nagpur, Maharashtra

## Abstract

**Introduction:**

Hematological abnormalities are a common complication of HIV infection. Bone marrow abnormalities occur in all stages of HIV infection. Present work was carried out to study the bone marrow abnormalities in patients with HIV/AIDS.

**Methods:**

160 patients of HIV +ve were included in the study. A complete blood count, relevant biochemical investigations, CD4 counts were done, besides a thorough history and clinical examination. HIV positive patients were classified as those having AIDS and those without AIDS according to NACO criteria. Bone marrow examination was performed for indication of anemia, leucopenia, pancytopenia and thrombocytopenia.

**Results:**

As per CDC criteria 59.81% patients had AIDS in 107 patients. The most common hematological abnormality was anemia, seen in 93.12% patients. Bone marrow was normocellular in 79.06% of non-AIDS and 79.68% of AIDS, hypocellular in 13.95% of non-AIDS and 12.5% of AIDS, hypercellular in 06.97% of non-AIDS and 07.81 % of AIDS patients. Dysplasia was statistically and significantly associated with anemia. For myelodysplasia in bone marrow in HIV patients we noted granulocytic dysplasia in 4.65% in Non – AIDS and 14.06% AIDS patients. Erythroid dysplasia was found in 9.30% in Non – AIDS, 12.5% in AIDS group. Thrombocytopenia was seen in 4 cases of ART (4.93%) and 3 cases (4.68%) of AIDS group. Abnormal cells like plasma cell, histiocyte and toxic granule were found in bone marrow.

**Conclusions:**

Myelodysplasia was more common in AIDS than in non AIDS patients. Granulocytic series is most commonly associated with evidence of dysplasia. Anemia in HIV patients can be a good clinical indicator to predict and access the underlying immune status. Thus bone marrow study is imperative to methodically observe and follow clinical and laboratory aberration in such patients in order to improve our diagnostic and therapeutic skills pertinent to HIV/AIDS.

## Introduction

HIV infection is multisystem disease and hematological abnormalities are among the most common complications of HIV. Bone marrow abnormalities are found at all stages of HIV disease, increasing in frequency as the disease progresses. Infection of marrow mesenchymal stem cells with HIV has been incriminated as an important factor causing bone marrow defects. A number of characteristic but nonspecific, morphologic abnormalities of the bone marrow of AIDS patients have been reported. Bone marrow examination may be useful for the definitive assessment of iron stores which can assist in the differentiation of iron-deficiency anemia from anemia of chronic disease. Bone marrow is a target for the combined effect of infection, drugs and chronic disease, 1) Cellularity of the bone marrow on trephine biopsy is usually normal or increased. 2) Dysplastic changes are common in erythroid and granulocytic lines. 3) Megaloblastic changes in the red cell series are seen and that may reflect myelodysplastic changes. 4) Plasma cell and histiocytes – often observed is likely to be repeated infection. 5) Reticuloendothelial iron block may seen in patients with AIDS is reflection of their clinical condition, with repeated episodes of infection. In Indian Study bone marrow, myelodysplasia was found to be 32.43% of HIV patients. Granulocytic series most commonly was associated with evidence of dysplasia. These dysplasia is common in patients with anemia (A.K. Tripathi, 2005)^1^. Study done by Donald S. et al (1990)^2^ showed correlation between morphologic finding and clinical/therapeutic feature/Laboratory finding, common bone marrow finding, hypercellularity 53%, myelodysplasia 69%, megaloblastic hematopoiesis 38%, plasma cytosis 25%, lymphocyte aggregate 36%. Other few studies showed dysgranulopoiesis to be more frequent and more accentuated than other land of dyserythropoiesis. This dyserythropoiesis may manifest as florid megaloblastic changes. Here, we aimed at studying the bone marrow abnormalities in patients with HIV disease who admitted to Government medical college and hospital and attending ART clinic. Both patients on ART and Non ART were included in the present study.

## Material and Methods

The study population included 160 HIV +ve symptomatic or asymptomatic patients. Out of that 139 males and 21 were females. Commonest age group involved was 21 to 40 years. HIV was diagnosed by ELISA method as per NACO guidelines. The study was conducted in Department of Medicine and Department of Pathology, Government medical college and hospital, Nagpur, Maharashtra.

Inclusion criteria: Indoor patients from medicine wards and those attending ART clinic included in the study.

Exclusion criteria: Patients of malignancy not related to HIV disease and patients receiving chemotherapy were excluded. Detailed history was taken which mainly included age, sex, place of residence, occupation, history of blood or blood product transfusions, high risk behavior, fever, weight loss, diarrhoea, oral or genital ulcerations, bleeding diathesis or history suggestive of systemic involvement. All patients were subjected to thorough physical examination both, systemic and general with necessary investigation like CD4 count, Hb%, CBC by cell counter, USG abdomen and CSF examination. Patients were classified into two clinical groups according to NACO criteria. 1. AIDS: (Those patients who fulfilled diagnostic criteria of AIDS according to NACO guidelines) 2. Non AIDS: (Asymptomatic and symptomatic, who did not fulfill the NACO AIDS criteria).

### Bone marrow study

Bone marrow examination was performed for indication of anemia, leucopenia, pancytopenia and thrombocytopenia. Posterior superior iliac spine was chosen as the site for bone marrow aspiration and biopsy because of large marrow space and least painful site. In obese and old patients, sternum was used for bone marrow aspiration. Smears of aspirated material were prepared immediately, dried and stained with Leishman Stain in hematology laboratory.

### Leishman’s Stain

Use commercially available stain or prepare stain as follows:

1. Add glass beads to 500 ml of methanol.
2. Add 1.5 g of Leishman’s powder.
3. Shake well, leave on a rotary shaker during the day then incubate at 37°C overnight. There is no need filter.

### Method

1. Make a thin film and air-dry rapidly.
2. Place the film on a staining rack, flood with Leishman’s stain and leave for 30 second to 1 min to fix.
3. Add a twice as much buffered distilled water, pH 7.2.
4. Leave to stain for 10 minutes.
5. Wash off stain with tap water.

Bone marrow sample was examined for cellularity, morphologic data including myeloid cell, erythroblast, megakaryocyte, lymphocyte, plasma cell, histiocyte, dysplastic changes, and fibrosis, granuloma and iron stores. Other investigations performed were hemoglobin, total leucocyte count, differential leucocyte counts, absolute neutrophil, lymphocyte, monocyte, eosinophil and basophil counts, general blood picture, platelet count, reticulocyte count, mean corpuscular volume, mean corpuscular hemoglobin, mean corpuscular hemoglobin concentration and total red blood cell count. CD4 count was done in 107 patients. Anemia was defined as hemoglobin <13 g/dl (Men) and <12 g/dl (women). Leucopenia was defined as total WBC count less than 4000 cells/μl. Neutropenia was defined as absolute neutrophil count <1000 cells/μl. Lymphopenia was considered when absolute lymphocyte count <800 cells/μl. Thrombocytopenia was defined as total platelet count < 150 × 103/μl. The study was carried out after obtaining permission from the Institute’s Ethics Committee. Also consent of patient or relative was taken.

## Result

Study title “Bone Marrow abnormalities in HIV disease” was conducted in Government Medical College and Hospital, Nagpur. Commonest age group involved, was 21 to 40 years (80%), similar findings were reported from other studies. There was a male preponderance with male to female ratio of 7:1; this may be because of more symptomatic male reporting to the hospital for testing HIV–positivity and treatment. Commonest population affected was that of drivers and laborers. Anemia (Hb % <10g/dl) was found in 93.12% cases, of this normocytic normochromic in 88.8% in Non-AIDS, 95.31%in AIDS group. In patients on ART bone marrow was hypercellular, hypocellular, and normocellular in 15(18.51%), 15(18.51%), and 51(62.96%) respectively. In non-ART group bone marrow was hypercellular, hypocellular, and normocellular in 7(8.86%), 7(8.86%), 65(82.27%) respectively. The bone marrow found normocellular in majority cases of non-ART group as compared to ART (p=0.130). In ART group bone marrow showed megaloblastic 8 patients (9.87%) and micronormoblastic 5 patients (6.17%), where as in Non-ART group 5 patients (6.32%) had megaloblastic bone marrow. Thrombocytopenia was seen in 4 cases of ART (4.93%) group and 3 cases (4.68%) AIDS group, in spite of normal to increase megakaryocytes in bone marrow suggesting auto immune mechanism for thrombocytopenia. Dysplasia was considered on the basis of anemia and myelodysplasia was noted in two cell line (erythroid 12.5% and granulocytic 14.06%). Patients having granulocytic dysplasia in the form of toxic granules or shift to left had infection like, gastroenteritis, pleural effusion and splenic abscess. Erythroid dysplasia was seen 12.5% in AIDS group was on HAART or Septran prophylacticaly. Increase histiocytes in bone marrow was seen in 2 cases Non-ART and ART group each.

## Observation

The data were analyzed using mean, standard deviation. A comparison between AIDS and Non-AIDS was done by chi-square test. A p value < 0.05 was taken as statistically significant.

## Discussion

Total 160 (139 males and 21females) HIV positive patients were included. These patients were divided into two groups’ on-ART and Non-ART with 81 and 79 patients respectively. CD4 count could be done in 107 out of 160 patients due to technical difficulty and financial problem. In that 64 patient’s CD4 counts <200/ul considered as AIDS according to the CDC criteria and 43 patients CD4 counts >200/ul considered as Non- AIDS (Table 6). Study done by A.K Tripathi et al (2005, Feb)^1^, included 74 HIV- positive patients with male to female ratio was 4:1,and the commonest age group was 20–40 years with range of 20 to 68 years. In our study we included total 160 HIV–positive patients with 139 males and 21 females(male to female ratio 7:1) most commonest age group in our study was 21 to 40 years, total 129(80.62%), range from12 years to 65 years (Table 1). We also categorized the patients according to occupation, most common population was of Drivers and Laborers, 54(33.75%) and 53(33.12%) respectively, Businessmen 13(8.13%), farmer 13(8.13%), government servant 5(3.12%), all 21(13.13%) females patients were house wives, and one 12 years student (Table 3). Patients were classified according to the duration of HIV positivity from history only and laboratory test reports (Table 4). In our study 40.00% of patients in AIDS (64 patients), while 26.87% in non–AIDS (43 patients), as we were able to do CD4 count only in 107 patients out of 160 patients. Anemia was one of the most common finding in HIV positive patients. Nora c j sun et al (1989)^3^ defined the following criteria in their study, anemia if hemoglobin concentration <10g/dl, leucopenia when white blood cell count < 4.0×10^9^/L and thrombocytopenia was defined as platelet count < 50×109/l. In present study we defined with hemoglobin concentration <10g/dl for anemia and that for leucopenia white blood cell count<4.0×10^9^/l, thrombocytopenia when platelet count < 100×10^3^/ul. Out of 160 patients 149 (93.12%) had hemoglobin % <10g/dl and 11 (6.87%) patients whose hemoglobin % > 10 g/dl. Hematological parameters in non-AIDS and AIDS group showed hemoglobin % in Non-AIDS 8.51±2.18 and in AIDS 8.86±1.51 (P = 0.3377). There was no statistical significance. Red blood cell (mill/mm^3^) 3.18±0.92, 3.51±0.84(NS), Mean corpuscular volume (fl) 78.18± 0.75,86.89±12.37, Mean corpuscular hemoglobin (pg) 23.56± 3.55,27.75±6.81Lymphocyte counts 1.06 ± 0.91,1.74±1.11, Granulocyte%70.04±1.08,64.75±10.11, Platelet counts (lac/mm^3^) 140.35± 67.01,189.01±74.67, CD4 counts/ul 290.51±79.90, 117.64±51.02 (Table 7), all these values showed statistically significant. Rests of the parameter were not showed any statistical significance as compared to previous. Bone marrow changes are often seen in HIV disease (Table 8). In ART group bone marrow normocellular in 51 patients, hypocellular marrow in 15 patients which was due to patchy involvement mostly by fatty degeneration of bone marrow. Hypercellular marrow in 15 patients because of erythroid hyperplesia. Whereas patients on Non- ART normocellular bone marrow in 65 patients. Hypocellular marrow was found in 7 patients, hypercellular in 7 patients. The difference between the ART and Non-ART group found to be statistically significant in patients of normocellular bone marrow (P= 0.000). Study by Donald S. et al (1991)^2^ showed marrow hyper cellularity 52% patients, hypocellularity in13% patients. Marrow cellularity was based on fat to cell ratio in relation to patients’ age. However in HIV–infected patients, hypercellularity was only appeared when there is increase number of non-hemopoietic cell such as lymphocyte, plasma cells and histiocytes. Tripathi A. K.(2005)^1^ studied on cellularity showed marrow in Non-AIDS normocellular 78.95%, hypocellular in 5.26%, hypercellular in 15.79% patients and in AIDS group normocellular in 74.5%, hypocellular in 7.27%, and hypercellular in 18.18% patients. Lionard et al (1987)^4^ conducted similar study, showed in group A(seropositive with active infection and drug therapy), normocellular 15%, hypocellular 8%, hypercellular 77%, while in group B(seropositive with no active infection and no drug), normocellular 0%, hypocellular 52%, hypercellular 48%. Gonzales et al (1995)^5^ showed only hypocellular bone marrow in 65% HIV positive patients. Nora et al (1989)^3^ showed bone marrow in AIDS, hypercellular 50% in AIDS, 31% in ARC. Jeery L. et al (1984)^6^ showed normocellular in 33.33%, hypocellular 25%, hypercellular in 66% patients. In our study we also found that most of the marrow was normocellular 79.43% patients in AIDS and non-AIDS, hypercellular in 7.39%, hypocellular in 13.22% in both the groups, this hypocellularity of bone marrow due serous fatty degenration. Majority of patients 65.62% were diagnosed to had HIV infection less than 1year, which was the cause for normocellular marrow as most of study showed hypercellular bone marrow. In ART group bone marrow showed normoblastic 68 patients, megaloblastic 8 patients (9.87%) and micronormoblastic 5 patients (6.17%). Where as in Non-ART group 5 patients (6.32%) had megaloblastic and micronormoblastic bone marrow and 69 patients had normoblastic. Thrombocytopenia associated with HIV infection has been described by Walsh et al (1984)^7^, Murphy et al (1987)^8^ suggests an autoimmune mechanism for the thrombocytopenia (30% AIDS) and neutropenia (20% AIDS), Lionard I. et al (1987)^4^ studied that majority of patients with thrombocytopenia adequate or increased megakaryocytic were evident in bone marrow. Jerry L. et al (1984)^6^ showed thrombocytopenia was initially in 3(25%) patient and subsequently developed in 2 others. Patwardhan M.S et al (2002)^6^ showed thrombocytopenia in 65 patients (13%) with average platelet count 0.92×10 (3)/ul. In our study thrombocytopenia on marrow showed increased megakaryocytes number secondary to increase peripheral distruction of platelet. In 4 patients of thrombocytopenia in ART, 2 patients had pulmonary tuberculosis and 2 had gastroenteritis. In these patients normoblastic bone marrow in 3, micronormoblastic in 1 patients. Myelodysplasia has been known to be with HIV infection for several years. Donald S. et al (1991)^2^ noted the myelodysplastic changes involving at least one cell line in 69% of their specimens, with erythroid, megakaryocytic, and granulocytic dysplasia in decreasing order (56%,31%and 18%respectively), A. K. Tripathi et al (2005)^1^ noted granulocytic, erythroid and megakaryocytic dysplasia 20%,3%,1% respectively in Non-AIDS and AIDS groups, Lionard I. et al (1986)^4^ showed myelodysplasia in group–A and group–B,( mention previously) Dysplasia of any type in 8(62%) out of 13 group A, 9(43%)out of 21in group B, erythroid 7 (54%) in group–A, 9((43%)in group B, myeloid dysplasia 5(38%) in group A and 5(24%)in group B. For myelodysplasia in bone marrow in HIV patients we noted granulocytic dysplasia in 4.65% in Non – AIDS, and 14.06% AIDS patients commonest infection found either tuberculosis or diarrhea. Erythrocytic dysplasia in 9.30% in Non–AIDS, 12.5% in AIDS group (Table 9). Commonest infection was found to be pulmonary tuberculosis and diarrhea, these patients also on HAART (Navirapine, zidovidine), while megakaryocytic dysplasia was not observed in both groups, in AIDS granulocytic changes in 9 patients and they had infection like, pulmonary tuberculosis and gastroenteritis, while in Non-AIDS 2 patients with pulmonary tuberculosis. Erythroid dysplasia was seen in 8 patients in AIDS group. Commonest infection was found to be tuberculosis and gastroenteritis which suggests that the myelodysplasia associated with infection probably was a direct result of the disease, which worsens with disease progression, and can be a side effect of therapy. We could not found any case of megakaryocyte dysplasia. We found abnormal cell in bone marrow 1) Plasma cell: In both groups, ART and Non ART total 18 (11.25%) patients out of 160 (5 patients ART, 13 patients Non–ART), infection in the patients of ART was pulmonary tuberculosis in1, gastroenteritis 3, bronchopneumonia 1 patients, while in Non ART, pleural effusion (tubercular) 1, gastroenteritis 3, oral candidiasis 4, meningitis 3(Crypto 2, TBM 1), splenic microabscess and urinary tract infection in 1 each. 2) Histocyte: found in 4 patients in both groups, 2 in each, Non–ART group 2 patients with tubercular pleural effusion, and ART group 2 patients with abdominal Koch’s. 3) Toxic granules: in 3 patients 1 on ART was pulmonary tuberculosis and 2 on Non-ART, in that pulmonary tuberculosis and gastroenteritis in 1 patient each.

## Limitation of Study

1) CD4 counts could not be done in all subjects due to technical problems. 2) We could get symptomatic HIV patients, so as to study hematological profile in early Stage of HIV infection. 3) No follow up was done as to review the hematological changes on HAART with improvement in CD4 counts.

## Figures and Tables

**Table 1:** Age distribution

Age groups.	No. of patients	%
12 to 20 yrs	3	1.8
21 to 30 yrs	60	37.6
31 to 40 yrs	69	43.12
41 to 50 yrs	22	13.75
51 to 60 yrs	4	4.5
61 and above	2	1.25

**Table 2:** Sex distribution.

Male	%	Female	%
139	86.87	21	13.12

**Table 3:** Occupation of patient

Occupation	No. of patients	%
Drivers	54	33.75
Laborer	53	33.12
Businessmen	21	13.13
Govt. servant	13	8.13
House wife	13	8.13
Farmer	5	3.12
Student	1	0.62
Total	160	100

**Table 4:** Duration of illness.

< 6 month	43
6 month to 1 year	62
>1 year to 2 year	23
> 2 year to3 year	15
> 3 year	17
Total	160

**Table 5:** Hemoglobin %.

HB%	No. of patients	%
5 to 8 gm%	43	26.87
Above 8 to 10 gm%	106	66.25
Above 10 to 14 gm%	11	6.87
Total	160	100

**Table 6:** CD4 counts.

CD4 counts	No. of patients	%
< 200 (AIDS)	64	59.81
200 To 500 (OI)	42	39.25
> 5oo (Non AIDS)	1	0.93

**Table 7:** Values and standard deviations of bone marrow parameters.

Hematological parameters	Non-AIDS Mean SD (n=43)	AIDS Mean ± SD(n=64)	P values
Hemoglobin% (g/dl)	8.51 ± 2.18	8.86 ± 1.51	0.3377 NS
Red blood cell count (million/mm^3^)	3.38 ± 0.92	3.51 ± 0.84	0.4232 NS
Mean corpuscular volume (MCV) (fl)	78.18 ± 0.75	86.89 ± 12.37	0.0001 HS
Mean corpuscular Hemoglobin (MCH)(pg)	23.56 ± 3.55	27.75 ± 6.81	0.0003 HS
Lymphocyte × 10^3^	1.06 ± 0.91	1.74 ± 1.11	0.001 HS
Granulocyte × 10^3^	3.82 ± 2.75	4.63 ± 1.98	0.0790 NS
Monocyte × 10^3^	0.41 ± 0.42	0.49 ± 0.2	0.2720 NS
Lymphocyte (%)	21.14 ± 11.43	25.65 ± 9.22	0.0265 S
Granulocyte (%)	70.04 ± 11.08	64.75 ± 10.11	0.0058 HS
Moncyte (%)	7.91 ± 4.35	9.87 ± 5.28	0.0454 S
Platelet count (lac/mm^3^)	140.35 ± 67.01	189.01 ± 74.67	0.0008 HS
CD4 count/ul	290.51 ± 79.90	117.64 ± 51.02	0.000 HS

(NS = Not significant, S= Significant, HS= Highly significant).

**Table 8:** Bone marrow cellularity in HIV/AIDS patients.

Bone marrow cellularity	Non-AIDS (n=43) %	AIDS (n=64) %	Total (n=107) %
Normocellular	34 (79.06)	51 (79.68)	85 (79.43)
Hypocellular	6 (13.95)	8 (12.5)	14 (13.22)
Hypercellular	3 (06.97)	5 (07.81)	08 (07.39)

**Table 9:** Myelodysplasia in bone marrow in HIV patients.

Dysplasia	Non-AIDS(43)		AIDS(64)	
	No.	%	No.	%

No dysplasia	37	86.04	49	76.56

Granulocytic	2	4.65	9	14.06

Erythroid	4	9.30	8	12.50

Megakaryocytic	0	0.0	0	0.0

Total	43	100	64	100

**Table 10:** Bone marrow cellularity findings in patients on ART and Non-ART.

Bone marrow	No. of patients (ART)	No. of patients (Non-RT)
Normocellular	51	65
Hypocellular	15	07
Hypocellular	15	07
Total	81	79

**Table 11:** Abnormal finding in bone marrow.

Abnormal cells	Bone marrow (no. of patients)		Total
	ART	Non-ART	
Plasma cell	5	13	18

Histocyte	2	2	4

Toxic granules	1	2	3
